# Adipokines in Sleep Disturbance and Metabolic Dysfunction: Insights from Network Analysis

**DOI:** 10.3390/clockssleep4030027

**Published:** 2022-06-22

**Authors:** Zhikui Wei, You Chen, Raghu P. Upender

**Affiliations:** 1 Department of Neurology, Vanderbilt University Medical Center, Nashville, TN 37232, USA; raghu.p.upender@vumc.org; 2 Department of Biomedical Informatics, Vanderbilt University Medical Center, Nashville, TN 37232, USA; you.chen@vumc.org

**Keywords:** adipokine, metabolic syndrome, sleep disorder, cardiovascular disease, network analysis

## Abstract

Adipokines are a growing group of secreted proteins that play important roles in obesity, sleep disturbance, and metabolic derangements. Due to the complex interplay between adipokines, sleep, and metabolic regulation, an integrated approach is required to better understand the significance of adipokines in these processes. In the present study, we created and analyzed a network of six adipokines and their molecular partners involved in sleep disturbance and metabolic dysregulation. This network represents information flow from regulatory factors, adipokines, and physiologic pathways to disease processes in metabolic dysfunction. Analyses using network metrics revealed that obesity and obstructive sleep apnea were major drivers for the sleep associated metabolic dysregulation. Two adipokines, leptin and adiponectin, were found to have higher degrees than other adipokines, indicating their central roles in the network. These adipokines signal through major metabolic pathways such as insulin signaling, inflammation, food intake, and energy expenditure, and exert their functions in cardiovascular, reproductive, and autoimmune diseases. Leptin, AMP activated protein kinase (AMPK), and fatty acid oxidation were found to have global influence in the network and represent potentially important interventional targets for metabolic and sleep disorders. These findings underscore the great potential of using network based approaches to identify new insights and pharmaceutical targets in metabolic and sleep disorders.

## 1. Introduction

Metabolic dysfunction and sleep disturbance are prevalent conditions in the modern obesity epidemic, leading to poor quality of life and increased morbidity and mortality [1,2]. Metabolic dysfunction, namely, obesity, insulin resistance, hypertension, and hyperlipidemia, also known as metabolic syndrome, is closely associated with sleep disturbance [3]. Despite the consistent associations in epidemiology studies, the underlying mechanisms of these associations remain poorly understood. Metabolic syndrome and sleep disorders such as obstructive sleep apnea (OSA) share similar risk factors, health outcomes, and biomarkers, making it difficult to tease out the cause and effect relationship [1]. Treatment of OSA also failed to show consistent evidence of improvement in metabolic profile unless accompanied by weight loss [4]. These observations indicate the presence of additional players in the interactions between sleep disturbance and metabolic dysfunction. Recent literature highlighted the visceral adipose tissue as a key predictor of both OSA and metabolic syndrome [5,6], prompting great interest in better understanding adipose tissue and adipose derived factors in these conditions. 

Adipokines are an ever-expanding group of peptide or protein hormones produced in the adipose tissue [7]. They are important messengers in relaying information between central nervous system, adipose tissue, and other metabolically active tissues and organs [8]. They also have vital roles in mediating complex interactions between metabolism, inflammation, and cardiovascular diseases [8]. Accumulating evidence suggests that adipokines may play a role in mediating the close association between the sleep disturbance and metabolic dysregulation as well [9]. Adipokines, such as leptin and adiponectin, have been shown to have multifaceted roles in sleep physiology and disorders. Sleep disturbance can also lead to changes in adipokines that contribute to systemic metabolic derangements [9]. Deep analysis of adipokines and their biological functions therefore represents an effective way of identifying new insights into the relationship between sleep disturbance and metabolic dysregulation.

Network analysis is a set of concepts and methods derived from graph theory. It is often used to visualize and study networks based on pairwise relationships [10]. Traditionally it is widely used to study social structure and networks [11]. However, it has been increasingly applied to biological systems due to the rapid generation of large scale “omics” data, necessitating systemic and integrative approach to extracting information [12]. Adipokines are excellent candidates for network analysis as they mediate complex crosstalk among a large array of biomolecules [13]. Their functions may be incompletely understood when the analysis is focused on a specific adipokine at a time. Further, additional insights can be obtained when adipokines and their targets are synthesized and analyzed in a network. The goal of the current study is to construct an adipokine network related to sleep disorders and metabolic dysfunction and use this network to explore new insights into the interplay between sleep disturbances, metabolic derangements, and relevant disease processes.

## 2. Basic Concepts in Network Analysis

**Figure 1:**
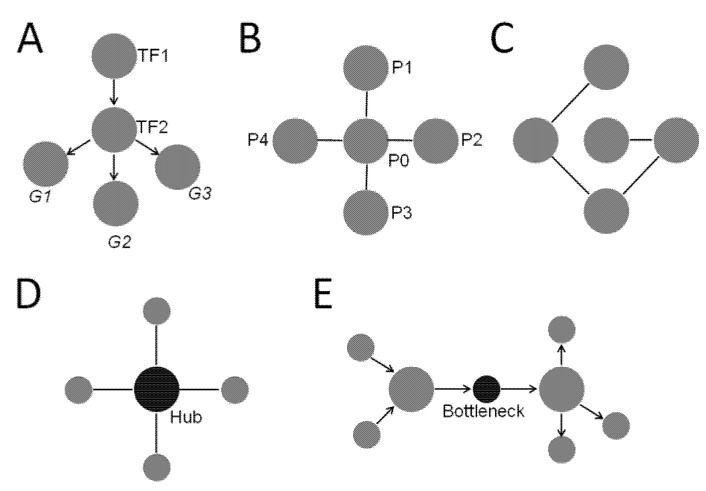
Representative networks and network metrics. (**A**) A hypothetical gene regulatory network showing nodes connected by directed edges. TF1 and TF2: Transcription factors 1 and 2. G1, G2, and G3: Genes 1, 2, and 3. (**B**) A hypothetical protein and protein interaction network showing nodes connected by undirected edges. P0, P1, P2, P3, and P4: Proteins 0, 1, 2, 3, and 4. (**C**) An example of a random network. (**D**) A network with the node of high degree centrality, referred as a “hub”, shown in black. (**E**) A network with the node of high betweenness centrality, referred as a “bottleneck”, shown in black.

Network analysis is a framework to visualize and analyze complex relations based on graph theory. A network (also known as a graph) only has two components—nodes and edges. The nodes in a biological network can represent entities such as genes, proteins, pathways, and diseases. Edges are linkage between two nodes that convey direct or indirect relations [10]. Directed edges convey a clear flow of information as seen in a gene regulatory network (Figure 1A). Undirected edges indicate close associations between two nodes without a direction of information flow. These can be seen in protein and protein interaction (PPI) networks (Figure 1B) [10].

### Network Metrics

The purpose of building a network is to obtain structure and relationship information from the network that would be otherwise unavailable if the components of the network were analyzed separately. Network analysis can be performed to obtain relevant network metrics. The metrics used in the current study include the following:

1. Degree—Degree is a fundamental metric of a network that determines many other aspects of the network. Degree of a node is simply the number of edges each node is connected to. For network with directed edges, each node may have an in-degree, which is the number of edges going into the node, and/or an out-degree, which is the number of edges coming out of the node [10]. Degree can be used to define other properties of a network, such as centrality.
2. Centrality—Centrality identifies the most “central” node of the network. However, being central may represent different properties in a network. For example, it could mean a node that is well-connected. That is, nodes with many edges connected to them are “central”. This is defined as degree centrality. The degree centrality of node *i* is calculated as $C_{i}=deg\left(i\right)$ where *deg(i)* is the node’s degree or edges [10]. Another important property of being “central” is a strategic position where the node lies in the cross paths of many shortest paths connecting other nodes. This is called betweenness centrality, defined as the extent to which a given node lies in the shortest paths connecting other nodes [10]. It is calculated as $C_{bet\left(i\right)}=\frac{\sigma_{xy\left(i\right)}}{\sigma_{xy}}$ where $\sigma_{xy}$ is the total number of shortest paths from node *x* to node *y* and $\sigma_{xy\left(i\right)}$ is the number of those paths that pass through node *i* [10]. A node with high betweenness lies on many shortest paths of other nodes which are not directly connected and has systems-level influence in the network (Figure 1; also see definition of bottlenecks below) [14,15].
3. Hubs—Hubs are nodes with much higher degrees than those of the other nodes in the network, although not every network has hubs. In a random network (Figure 1C), every node is connected to a low number of nodes, and all have similar degrees. Therefore, there are no hubs in a random network. In a scale-free network, most nodes are connected to a small number of high degree nodes that are considered as hubs (Figure 1D). It turns out most biological networks have properties similar to a scale-free network, which has a “small word phenomenon” by which distances between nodes are shortened significantly by the presence of hubs. In biological systems, hub proteins/genes are enriched in essential/lethal genes, and are often evolutionarily conserved [10].
4. Bottlenecks—Nodes with high betweenness centrality are known as bottlenecks (Figure 1E) [14]. They are similar to major highways in a transportation network with many shortest paths going through them (Figure 1E). Not surprisingly, bottleneck nodes control the information flow in a directed network, and disruption at these nodes causes widespread damage. In biological networks, bottleneck nodes are enriched in proteins/genes that are important for systems-level of phenotypes such as essentiality and virulence [15].

## 3. Methods

### 3.1. Construction of an Adipokine Network

In order to construct a network, one needs to identify the nodes and edges. In the current adipokine network, the nodes are selected adipokines with demonstrated functions in sleep physiology and disorder [9], and their interacting partners, such as molecular targets, and relevant physiologic functions and disease processes (Table S1). The edges are constructed as directed linkage among the nodes, which reflect current understanding of the molecular mechanisms of these adipokines based on following reviews [8,16,17,18,19,20,21]. These reviews were chosen due to their comprehensive presentation of molecular pathways and physiologic functions of these adipokines and extensive referencing.

The connections between the six types of nodes in the adipokine network are created as follows:

1. Suppose a physiologic perturbation or sleep disturbance has a positive/negative/uncertain association with an adipokine. In that case, a directed edge is created pointing from the perturbation/sleep disturbance to the adipokine with a label depicting a positive, negative, or uncertain relation. For instance, OSA is negatively associated with leptin level, so an edge from OSA to leptin is created with an edge label set as negative.
2. Edges are then created starting from each of the adipokine to their molecular targets. For instance, the Janus kinase–signal transducer and activator of transcription 3 (JAK–STAT3) pathway is one of the molecular targets of leptin, so an edge from the leptin to the JAK–STAT3 is created. Note that the edge between an adipokine and its molecular target does not imply physical interactions between the two molecules, but rather information flow from one to the other.
3. We then create the edge from a molecular target to a physiologic function of an adipokine if there is an association between them. For instance, the edge is created from AMPK to insulin signaling to indicate the information flow from AMPK to insulin signaling.
4. If a physiologic function is associated with a disease process, then an edge starting from the physiologic function to the disease process is created. For instance, the edge from insulin signaling to metabolic syndrome is created to indicate the close association between insulin signaling and metabolic syndrome.
5. We also create edges from an adipokine to its associated physiologic functions/diseases without identified molecular targets. For instance, leptin is associated with insulin signaling and metabolic syndrome. Thus, edges from leptin to insulin signaling and metabolic syndrome are created. This is to make sure the network has good “coverage”, even though some of the molecular targets/mechanisms of the linkage are unknown.

We performed the above 5 steps iteratively for each adipokine and their molecular targets to identify all the nodes and their edges in the adipokine work. All the nodes contained in the network are summarized in the Table S1. The network is then visualized and analyzed using an open-source network analysis platform “Gephi” [22] to derive network metrics.

### 3.2. Degree Centrality Analysis

Degree of a node was calculated using the equation $C_{i}=deg\left(i\right)$ where *deg*(*i*) is the node’s degree or edges. A node may have either an outdegree, indegree, or both depending on whether there are edges coming out of or going into the node or both. The degrees of the nodes were ranked from high to low to identify nodes with high degree centrality, which are considered hubs in a network.

### 3.3. Betweenness Centrality Analysis

Betweenness of a node was calculated using the equation $C_{bet\left(i\right)}=\frac{\sigma_{xy\left(i\right)}}{\sigma_{xy}}$ where $\sigma_{xy}$ is the total number of shortest paths from node *x* to node *y* and $\sigma_{xy\left(i\right)}$ is the number of those paths that pass through node *i.* The betweenness of the nodes was ranked from high to low to identify nodes with high betweenness centrality, which are considered bottlenecks in a network.

## 4. Results

### 4.1. General Characteristics of the Adipokine Network

We identified 94 nodes and 264 edges connecting the nodes in the adipokine network. The network is shown in Figure S1. Note that nodes with inconsistent/contradicting relationships from previous studies were excluded from the analysis. It has the following characteristics:

1. Directed edges—The edges between two nodes are directed representing signal flow from one node to another.
2. Weighted nodes—The nodes are weighted proportionally based on their degrees.
3. Scale-free network—Most nodes in the network are connected to a small number of nodes which have higher degree than those of the rest of the network. The nodes with high degree centrality are considered hubs, which are important members of a network.

This adipokine network depicts in the information flow from physiologic perturbations and sleep disturbance to disease processes, as shown in Figure S2. It also reflects the role of adipokines as important messengers in mediating systemic changes in metabolic diseases in response to physiologic stimuli [8].

### 4.2. Analysis of the Local Environment of the Adipokine Network

One challenge associated with analyzing adipokines is the complexity of relationships between adipokines, their molecular targets, and the metabolic pathways they act upon. It is often difficult to determine which molecules or signaling pathways are of particular importance for the network as a whole. Network analysis may be helpful in addressing such problems. There are a set of tools in network analysis known as centrality analyses, which identify the nodes that are of particular importance in a network. A simple and effective technique among them is the degree centrality, or degree for short. The degree analysis only considers the immediate neighbors of each node and therefore is a measure of local environment of the network.

**Figure 2:**
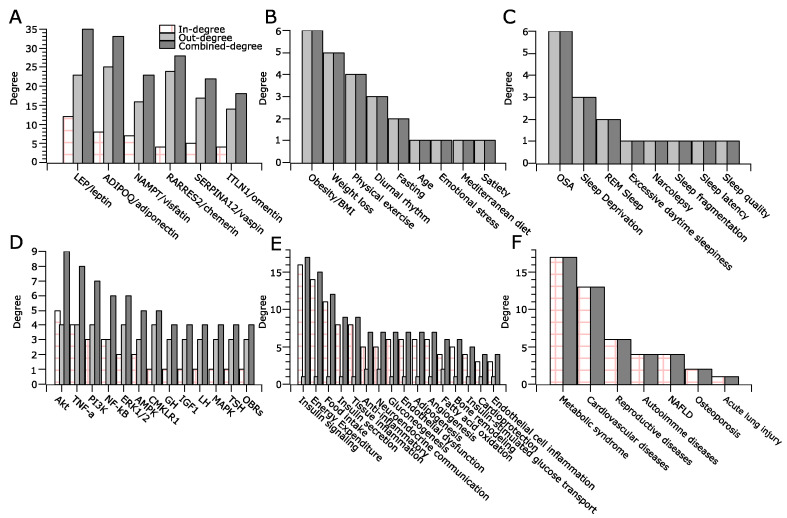
Degree analysis of the adipokine network representing adipokines (**A**), physiologic perturbations (**B**), sleep disturbances (**C**), molecular targets (**D**), physiologic functions (**E**), and relevant disease processes (**F**). Among them, physiologic perturbations (**B**) and sleep disturbances (**C**) only have out-degree, and disease processes (**F**) only have in-degree. Adipokines (**A**), molecular targets (**D**), and physiologic functions (**E**) have in-degree, out-degree, and combined degrees. Only selected molecular targets and physiologic functions are shown.

The degree centrality analyses were carried out for adipokines (Figure 2A), physiologic perturbations (Figure 2B), sleep disturbances (Figure 2C), molecular targets (Figure 2D), physiologic functions (Figure 2E), and disease processes associated with adipokines (Figure 2F). As shown in Figure 2A adipokines have higher degrees than other types of nodes in the network, and leptin and adiponectin having higher degrees than the rest of the adipokines. This suggests that adipokines, particularly leptin and adiponectin, serve as hubs of the network connecting physiologic and sleep disturbance to downstream molecular targets and pathways. As shown in Figure 2B,C, obesity and OSA are major drivers for the downstream changes mediated by adipokines, both of which are also important risk factors for metabolic dysregulation and development of metabolic disorders [23]. Figure 2D–F demonstrates that in response to physiologic perturbations and sleep disturbances, adipokines act on multiple signaling pathways that affect diverse physiologic functions including insulin signaling, inflammation, energy expenditure, and food intake that contribute to metabolic syndrome and cardiovascular diseases, among others. These results underscore the pleiotropic effects of adipokines in metabolic regulation [7]. In the meantime, Figure 2F suggests that roles of adipokines extends beyond cardiovascular disease and also involves other disease processes, such as reproductive and autoimmune diseases.

### 4.3. Analysis of Global Relationships in the Adipokine Network

Another important application of network analysis is to identify nodes that are critical in controlling the communication across the network. This is measured by betweenness centrality, or betweenness for short. Nodes with high betweenness are also known as bottlenecks, in the sense that disruption at these nodes can lead to widespread cessation of information flow, similar to a traffic jam at a major highway [14]. Therefore, betweenness centrality analysis is used here to identify nodes with global significance in the adipokine network. 

**Figure 3:**
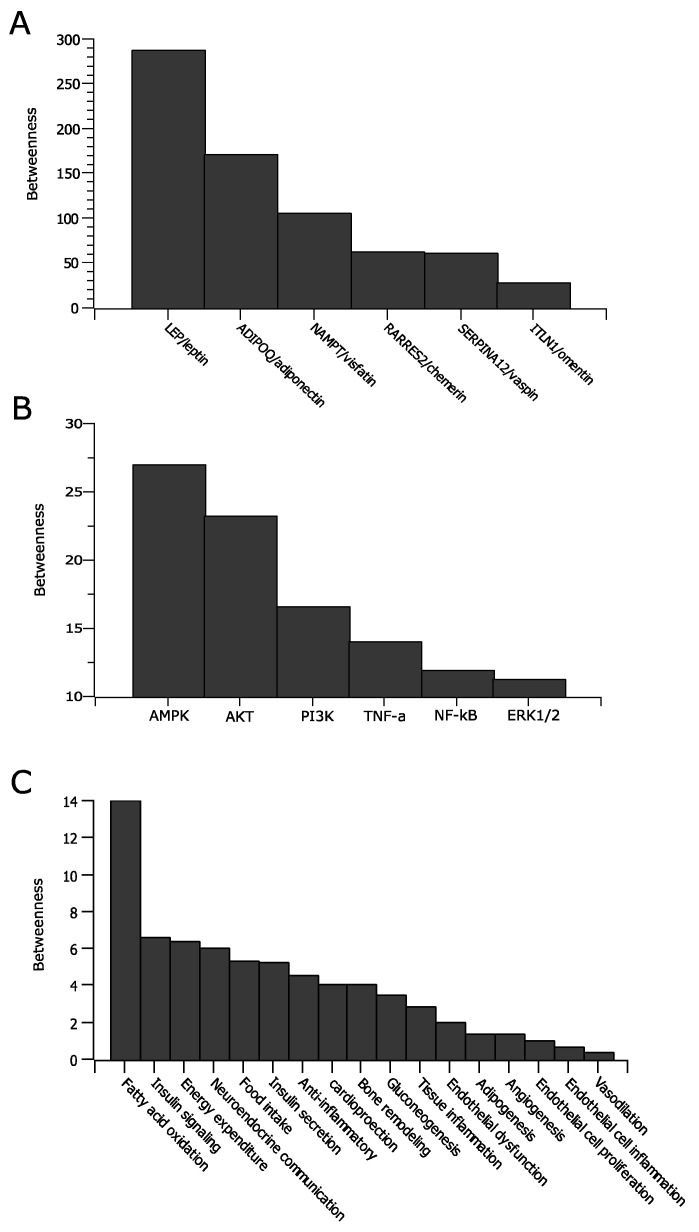
Betweenness centrality of the adipokine network, representing adipokines (**A**), molecular targets (**B**), and physiologic functions (**C**). Only selected molecular targets and physiologic functions are shown.

The betweenness of the adipokine network is shown in Figure 3; only nodes representing adipokines, molecular targets, and physiologic functions are shown, as those nodes are in paths connecting others (Figure S2). As shown in Figure 3A, leptin has the highest betweenness among the adipokines, highlighting its strategic position in mediating global metabolic changes associated with sleep and physiologic perturbations. This is consistent with a previous study showing that leptin pathway reflects a key mechanism with modifiable effects underlying sleep disturbance and obesity in children [24]. In Figure 3B,C, AMPK and fatty acid oxidation have higher betweenness than other molecular targets or metabolic pathways. This is because AMPK is connected to two adipokines, leptin and adiponectin, which are connected to majority of nodes in the network, and fatty acid oxidation is connected to AMPK and adiponectin. This finding is particularly interesting as AMPK and fatty acid oxidation do not have higher degrees compared with other targets or pathways (Figure 2D,E). These nodes are sometimes referred as nonhub bottlenecks, meaning nodes with low degree but high betweenness [14]. Nonhub bottlenecks have specific characteristics in biologic networks. For example, in a gene regulatory network, nonhub bottlenecks can represent key regulatory proteins such as transcription factors that confer essentiality for survival [14]. In the adipokine network, it is likely that they represent important points of regulation for the signaling across the adipokine network. Indeed, previous studies have shown that AMPK is a conserved cellular energy–sensing mechanism that plays fundamental roles in glucose metabolism, fatty acid oxidation, and protein synthesis. It has been implicated in the mechanisms of action for several important drugs in metabolic syndrome, including metformin, thiazolidinediones (TZDs), and statins [25]. It has been considered an important target for therapeutic interventions for cardiometabolic diseases [25]. 

## 5. Discussion

The relationship between sleep disorders and metabolic dysregulation has been intensely studied in recent years. This is mainly in the setting of modern epidemic of metabolic syndrome, a cluster of conditions include obesity, insulin resistance, hypertension, and hyperlipidemia. Metabolic syndrome leads to increased risk of cardiovascular disease and stroke and contributes to significant mortality and morbidity in modern society [3]. Clinical studies have shown that metabolic syndrome and sleep disturbance are closely related to each other. For example, metabolic syndrome is highly prevalent among patients with obstructive sleep apnea with rates ranging from >50% to >80% in different studies [3]. Meanwhile, the prevalence of OSA in patients with metabolic syndrome is almost 70% [26]. In addition to OSA, other sleep disturbance, such as sleep deprivation also plays significant roles in metabolic dysfunction. For instance, short sleep time in children has been linked to development of obesity in longitudinal prospective studies indicating a potential causal relationship [27]. The underlying mechanisms linking sleep disturbance and metabolic dysregulation are yet to be determined. Potential etiologies include sympathetic activation, systemic inflammation, and insulin resistance [28]. 

Accumulating evidence suggested that adipokines may also play a role in mediating the association between sleep disturbance and metabolic dysfunction [9,29]. One representative of such adipokines is leptin, which has demonstrated roles in improving upper airway airflow and hypopnea during sleep [30]. Leptin deficiency also results in significant changes in sleep structure, sleep duration, and circadian rhythmicity [31,32]. In the meantime, leptin has significant metabolic functions in satiety, immune responses, and insulin sensitivity [17]. Several other adipokines such as adiponectin are differentially regulated in sleep disorders and contribute to systemic metabolic dysfunction [9,29]. Indeed, the degree centrality analysis of the adipokine network indicated that obesity and sleep disturbance can impact multiple adipokines with more convergent functions on insulin signaling, energy expenditure, food intake, and tissue inflammation compared with other molecular and cellular processes (Figure 2). This finding highlights important channels of communication by these adipokines in systemic metabolic dysfunction. The versatile yet convergent functions of adipokines suggest the presence of network effects among these adipokines and their interacting partners in systemic metabolic dysfunction [13]. 

There is increased appreciation for network effects in the pathogenesis of diseases. This is based on the understanding that symptoms of a complex disease are unlikely to be the product of a single gene or molecular product but the sum of various pathophysiologic processes that interact in a complex network [33]. A better understanding of these disease networks may lead to identification of high impact pharmaceutical or interventional targets [33]. Previous research on biological networks has established important concepts in identifying these targets using centrality analysis. For instance, it was found that hub proteins tend to be conserved essential genes whereas bottleneck proteins tend to have systems-level functionality. In the current study, adipokines were found to serve as hubs in the network mediating complex processes that lead to diverse metabolic changes in response to obesity and OSA. Leptin, AMPK, and fatty acid oxidations were found to be the bottlenecks in the network that have global influence in the pathogenesis of metabolic dysfunction associated with sleep and physiologic perturbations. These findings underscore the great potential of using network-based approaches to identify new mechanisms or targets important for metabolic diseases.

There is a paucity of literature on network analysis of adipokines in metabolic diseases, providing an opportunity for further research. Xiuping Chen created four networks of adiponectin-related drug targets based on known and predicted PPI datasets. He identified a few molecules that could play important roles in the biological activities of adiponectin, which may serve as potential druggable targets [34]. Mechanick et al. created an adipokine–cardiovascular–lifestyle network based on evidence in selected expert reviews and a PPI network, highlighting the roles of adipokine subnetworks in clinical translations of cardiovascular diseases such as “obesity paradox”, “metabolically healthy obese”, and life style interventions [13]. Huang et al. constructed a correlation network of 27 biomarkers for type 2 diabetes using dataset from a case control study and revealed early perturbations of biomarker correlation structure years before clinical diagnosis of diabetes and the central role of leptin system in mid and late stages of diabetes [35]. Zhang et al. conducted a PPI and modular network analysis of adipose specific genes based on adipose tissue transcriptome and found two significant modules, one of which is significantly enriched in AMPK, peroxisome proliferator-activated receptor (PPAR), and insulin signaling [36]. While these studies serve as the foundation for future research on network analysis of adipokines, the present study represents a step in understanding the adipokine network effect in sleep disturbance associated metabolic dysfunction and relevant disease processes.

The network analyses performed in this research are based on published studies on adipokines and their function in sleep and metabolic disorders. An advantage of such an approach is that relationships between nodes are verified by independent functional studies rather than inferred from gene or protein libraries. This approach lowers the “noise” often seen in large-scale PPI networks that have abundant nonselected and nonfunctional interactions [37]. Nonetheless, it is worth noting that publication bias is intrinsic to such analysis. One consequence of the publication bias is that understanding of newer, less well-known adipokines and their roles in sleep and metabolic dysfunction is quite limited compared to those of leptin [17,38] or adiponectin [16]. The roles of individual adipokines in the network may change once new information is uncovered. New hub or bottleneck proteins could be identified as the adipokine network continues to evolve. Another limitation of the study is the need of an independent verification of the adipokine network by experimental or clinical data. Future large scale prospective studies and additional mechanistic studies are needed to further clarify the complex interplay between sleep and metabolic dysfunction mediated by the adipokine network.

## 6. Conclusions

Adipokines likely function in a complex network to play important roles in obesity, sleep disturbance, and metabolic dysfunction. The adipokine network acts on a wide array of molecular targets and physiologic pathways, particularly insulin signaling, energy expenditure, food intake, and tissue inflammation to regulate systemic metabolic changes in response to obesity, OSA, and other factors. A few additional interesting findings from the adipokine network analysis included: (1) Adipokines, particularly leptin and adiponectin, serve as hubs in the network that act in an ensemble to mediate the systemic metabolic changes in response to physiologic perturbations and sleep disturbances. (2) Leptin, AMPK, and fatty acid oxidation are bottlenecks in the adipokine network that have global influence in systemic metabolic dysfunction. These proteins and pathway have the potential to be important targets for the therapeutic intervention for sleep and metabolic disorders. 

## Supplementary Materials

The following supporting information can be downloaded at: https://www.mdpi.com/article/10.3390/clockssleep4030027/s1. Table S1. Adipokines, their molecular targets, and physiologic functions in metabolic diseases. (+): Positive correlation. (−): Negative correlation. (?): Unclear correlation. Common names and gene symbols for adipokines are provided. Diseases are listed in categories that may contain a number of related medical conditions. List of abbreviations: CVD: cardiovascular diseases. DM1: diabetes mellitus type 1. DM2: diabetes mellitus type 2. IBD: inflammatory bowel disease. HLD: hyperlipidemia. HTN: hypertension. NAFLD: non-alcoholic fatty liver disease. OSAS: obstructive sleep apnea syndrome. PCOS: polycystic ovarian syndrome. RA: rheumatoid arthritis. References for each adipokine and its biological functions are provided in the table. Figure S1. Global adipokine network: Adipokines and associated physiologic perturbations, sleep disturbances, molecular targets, physiologic functions, and relevant disease processes. The colors of the edges indicate the positive (green), negative (orange), uncertain (blue), or neutral (purple) relationships between any two nodes. The colors of the nodes represent adipokines (pink), physiologic perturbations (blue), sleep disturbances (brown), molecular targets (light purple), physiologic functions (light green), and metabolic diseases (dark green). Figure S2. Information flow in the constructed adipokine network.
