# Supplementary material for: Adipokines in Sleep Disturbance and Metabolic Dysfunction: Insights from Network Analysis
Source: Clocks Sleep. 2022 Jun 22;4(3):321–31. doi: 10.3390/clockssleep4030027 (PMC9326621; doi:10.3390/clockssleep4030027)
Supplement: Supplementary file 1 [file clockssleep-04-00027-s001.zip › clockssleep-1732004-supplementary.pdf]

Supplementary Materials

Table S1. Adipokines, their molecular targets, and physiologic functions in metabolic diseases. (+): Positive correlation. (-): Negative correlation. (?): Unclear correlation. Common names and gene symbols for adipokines are provided. Diseases are listed in categories that may contain a number of related medical conditions. List of abbreviations: CVD: cardiovascular diseases. DM1: diabetes mellitus type 1. DM2: diabetes mellitus type 2. IBD: inflammatory bowel disease. HLD: hyperlipidemia. HTN: hypertension. NAFLD: non-alcoholic fatty liver disease. OSAS: obstructive sleep apnea syndrome. PCOS: polycystic ovarian syndrome. RA: rheumatoid arthritis. References for each adipokine and its biological functions are provided in the table.

**Adipokines**	**Physiologic Perturbations**	**Sleep Disturbances**	**Molecular Targets**	**Physiologic Functions**	**Disease Processes**
LEP/Leptin [9,17,38]	Diurnal rhythm Emotional stress (+), Obesity/BMI (+), Satiety (+), Fasting (-) Physical exercises (-) Weight loss (-),	OSAS (+), Sleep quality (+), Excessive daytime sleepiness (-), REM sleep (-), Sleep deprivation (?)	Receptors: ObRs; JAK–STAT3, melanocortin, AgRP, NPY, POMC, CART; TSH, LH, GH, IGF1; PI3K, Akt, AMPK	Bone remolding, Energy expenditure, Fertility, Food intake, Insulin signaling, Neuroendocrine communication	Metabolic syndrome: HTN, obesity, insulin resistance, HLD; Osteoporosis; Reproductive: amenorrhea, infertility, PCOS
ADIPOQ/Adiponectin [9,16]	Age (+), Mediterranean diet (+), Physical exercises (+), Weight loss (+), Obesity/BMI (-)	OSAS (-), Sleep deprivation (?) Sleep fragmentation (-)	Receptors: AdipoR1, AdipoR2, T-cadherin; APPL1, IRS, PI3K, Akt, PEPCK, G6P; AMPK, PPAR-α; CRH; TNF-α	Antiapoptotic, Anti-inflammatory, Endothelial dysfunction, Anti-atherosclerotic, Cardioprotection, Energy expenditure, Fatty acid oxidation, Insulin signaling, Gluconeogenesis, Insulin-stimulated glucose transport	Atherosclerosis, CVD; Metabolic syndrome: HTN, obesity, insulin resistance, HLD; NAFLD
RARRES2/Chemerin [9,21]	Obesity/BMI (+), Physical exercise (-) Weight loss (-),	OSAS (+)	Receptors: CMKLR1, GPR1, Ccrl2; MAPK, ERK1/2, PI3K, Akt; GPR1; PGC1 α, G6P, PEPCK; TNF-α, IL- 1β	Adipogenesis, Angiogenesis, Chemotaxis, Gluconeogenesis, Insulin secretion, Insulin signaling, Insulin-stimulated glucose transport, Tissue inflammation	Autoimmune: IBD, RA; Atherosclerosis, CVD; Metabolic syndrome: obesity, insulin resistance, HTN, DM2, HLD; Reproductive: PCOS
		OSAS (?)	IR, Akt, KLK7;		Autoimmune: RA;

SERPINA12/Vaspin [9,19]	Diurnal rhythm Obesity/BMI (+), Fasting (-), Weight loss (-),		GRP78/VDAC complex; TNF-α, NF-κB ; NPY, POMC	Endothelial cell inflammation, Endothelial cell proliferation, Food intake, Insulin signaling, Vascular smooth muscle cell proliferation	Atherosclerosis, diabetic vascular diseases; Metabolic syndrome: obesity, insulin resistance, DM2; Reproductive: PCOS
ITLN1/Omentin [9,18]	Physical exercise (+), Weight loss (+), Obesity/BMI (-)	OSAS (?)	Akt; CRP, TNF-α, NF-κB; eNOS	Insulin signaling, Insulin-stimulated glucose transport, Endothelial dysfunction, Vasodilation	Autoimmune: Crohn's (IBD), RA, DM1; Metabolic syndrome: HTN, insulin resistance, DM2; NAFLD Reproductive: PCOS
NAMPT/Visfatin [9,20]	Diurnal rhythm Obesity/BMI (+)	Narcolepsy (+), Sleep deprivation (+), Sleep latency (+), REM sleep (-) OSAS (?)	IR, ERK1/2; Syntaxin 4, HNF4 α, HNF1β, NAD; NF-κB	Endothelial dysfunction, Insulin secretion, Insulin signaling, Insulin-stimulated glucose transport, Vascular smooth muscle dysfunction	Acute lung injury; CVD, atherosclerosis; Metabolic syndrome: obesity; NAFLD

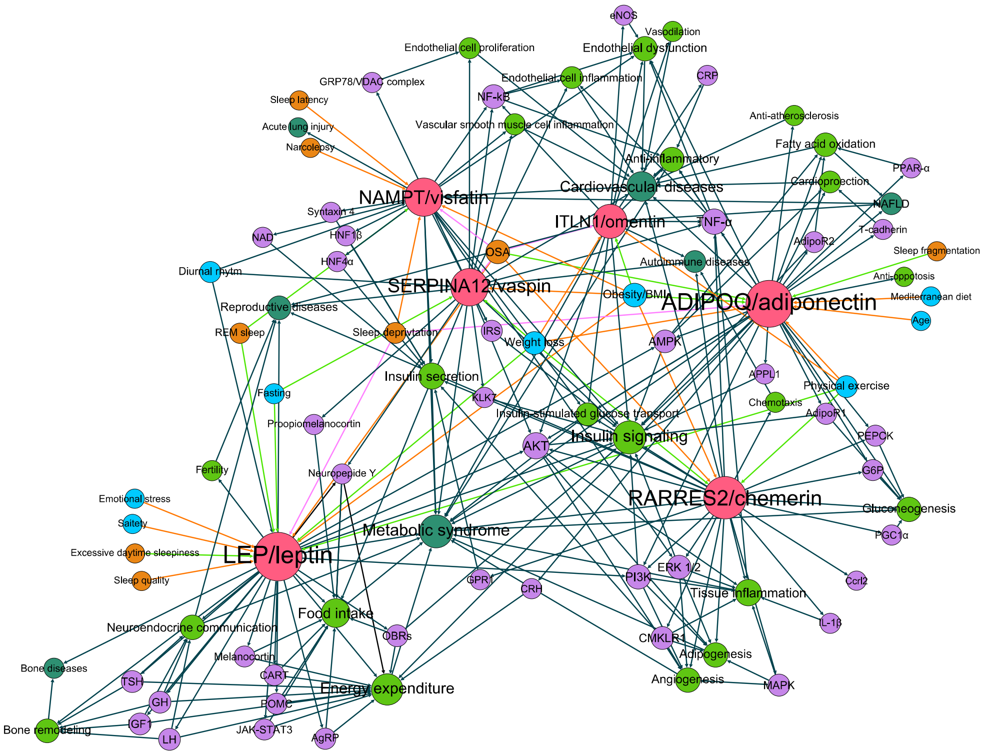

Figure S1. Global adipokine network: Adipokines and associated physiologic perturbations, sleep disturbances, molecular targets, physiologic functions, and relevant disease processes. The colors of the edges indicate the positive (green), negative (orange), uncertain (blue), or neutral (purple) relationships between

any two nodes. The colors of the nodes represent adipokines (pink), physiologic perturbations (blue), sleep disturbances (brown), molecular targets (light purple), physiologic functions (light green), and metabolic diseases (dark green).

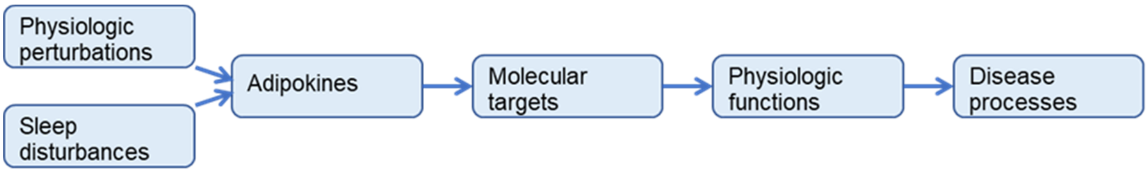

Figure S2. Information flow in the constructed adipokine network.
